# Optimal visceral adipose tissue thresholds that minimize risk for osteoporosis and/or diabetes in midlife women

**DOI:** 10.1210/jendso/bvag049

**Published:** 2026-03-06

**Authors:** Sheryl Yen Pin Tan, Kothandaraman Narasimhan, Beverly Wen Xin Wong, Liang Shen, Darren Yuen Zhang Tan, Johan Gunnar Eriksson, Eu-Leong Yong

**Affiliations:** Department of Obstetrics and Gynaecology, Yong Loo Lin School of Medicine, National University of Singapore, Singapore 119288, Singapore; Institute for Human Development and Potential, Agency for Science, Technology and Research, Brenner Centre for Molecular Medicine, Singapore 117609, Singapore; Department of Obstetrics and Gynaecology, Yong Loo Lin School of Medicine, National University of Singapore, Singapore 119288, Singapore; Biostatistics Unit, Yong Loo Lin School of Medicine, National University of Singapore, Singapore 117549, Singapore; Department of Obstetrics and Gynaecology, Yong Loo Lin School of Medicine, National University of Singapore, Singapore 119288, Singapore; Department of Obstetrics and Gynaecology, Yong Loo Lin School of Medicine, National University of Singapore, Singapore 119288, Singapore; Institute for Human Development and Potential, Agency for Science, Technology and Research, Brenner Centre for Molecular Medicine, Singapore 117609, Singapore; Folkhalsan Research Center, Helsinki 00250, Finland; Department of General Practice and Primary Health Care, University of Helsinki and Helsinki University Hospital, Helsinki 00014, Finland; Department of Obstetrics and Gynaecology, Yong Loo Lin School of Medicine, National University of Singapore, Singapore 119288, Singapore

**Keywords:** visceral adipose tissue, diabetes, osteoporosis, risk stratification, midlife women

## Abstract

**Context:**

Visceral adipose tissue (VAT) is increasingly recognized as a key determinant of diabetes and osteoporosis risk. However, clinically relevant VAT thresholds associated with both osteoporosis and type 2 diabetes remain undefined.

**Objective:**

Utilizing the diametrically opposing effects of VAT for risk of diabetes and osteoporosis, this study aimed to identify a healthy VAT range minimizing risk for both conditions.

**Methods:**

We used baseline data from the Integrated Women's Health Programme, a cohort of community-dwelling midlife Singaporean women, to construct a spline-based odds ratio (OR) curve to assess the nonlinear relationship between VAT and the combined risk of osteoporosis and/or diabetes. A healthy VAT range was defined as the interval where the OR approached 1.0. Validation was conducted using longitudinal follow-up over 6.8 years, with modified Poisson regression models adjusted for age and ethnicity to estimate relative risks for incident (new) disease.

**Results:**

In midlife women (n = 1185, mean age: 56.3 ± 6.2, age range: 45.0 to 69.6 years), a U-shaped spline OR curve for the combined risk of osteoporosis and/or diabetes was observed. An optimal VAT range of 46.5 to 116.6 cm^2^ was identified where the OR was ≤1.0 for both conditions. Participants with VAT <46.5 cm^2^ had a 2-fold increased risk of developing incident osteoporosis [adjusted relative risk (aRR): 2.04, 95% confidence interval (CI): 0.95-4.40] but no incident diabetes. Conversely, those with VAT >116.6 cm^2^ had a significantly higher risk of incident diabetes (aRR: 2.89, 95% CI: 1.68-4.98) and a lower risk of incident osteoporosis (aRR: 0.33, 95% CI: 0.17-0.64).

**Conclusion:**

These findings establish a clinically actionable VAT range (46.5-116.6 cm^2^) that may guide metabolic and skeletal disease risk stratification and inform preventive care strategies.

The rising obesity rate has been a major contributor to the global epidemic of type 2 diabetes mellitus (hereafter, “diabetes”) [1]. However, individuals with similar body mass index (BMI) exhibit markedly different diabetes risk profiles due to differences in fat distribution, particularly ectopic fat deposition [2-5]. This variability is especially pronounced in Asian populations, where individuals with “normal” BMI presented with diabetes risks comparable to obese individuals—a phenomenon termed the “skinny-fat” syndrome or the Asian phenotype [6, 7]. Recent studies have implicated visceral adipose tissue (VAT), as opposed to subcutaneous fat, as a key determinant of this paradoxical risk [8, 9].

In the UK Biobank cohort, VAT was positively associated with diabetes risk, whereas abdominal subcutaneous fat and gluteo-femoral fat showed neutral or protective effects [10, 11]. In lean individuals, disproportionate VAT accumulation may reflect genetic predispositions toward preferential visceral fat storage and evolutionary-selected metabolic adaptations that originally conferred survival advantages during periods of food scarcity but now predispose to metabolic dysfunction in environments of caloric abundance [12]. Although VAT has been linked to diabetes, clinically actionable thresholds have not been established [9]. Establishing such thresholds could enhance risk stratification and guide prevention strategies—especially given the emergence of VAT-targeting interventions, such as glucagon-like peptide-1 receptor agonists [13].

Osteoporosis, characterized by low bone mineral density (BMD) and compromised bone microarchitecture, has remained a leading contributor to morbidity and healthcare costs due to fracture-related disability [14]. By 2025, hip fractures are projected to exceed 21 million cases globally, with associated costs surpassing $25 billion [14, 15]. Although higher BMI has been thought to promote bone formation through mechanical loading, emerging data suggest VAT may exert independent and complex effects on bone [16, 17]. Large-cohort studies have demonstrated that low VAT is associated with lower BMD in postmenopausal women [18, 19]. These findings imply VAT has opposing effects on metabolic and skeletal health. High VAT raises diabetes risk [11, 20] but reduces osteoporosis risk, while low VAT lowers diabetes risk but increases osteoporosis risk [21].

Our study aims to, first, define VAT thresholds associated with the lowest risk of osteoporosis and/or diabetes cross-sectionally, and second, validate these thresholds for predicting incident disease. We hypothesize that a healthy VAT range can be derived from thresholds that define the lowest risk for both osteoporosis and/or diabetes, given that VAT has divergent effects on the risk for these 2 conditions.

## Methods

### Study setting and participants

**Figure 1:**
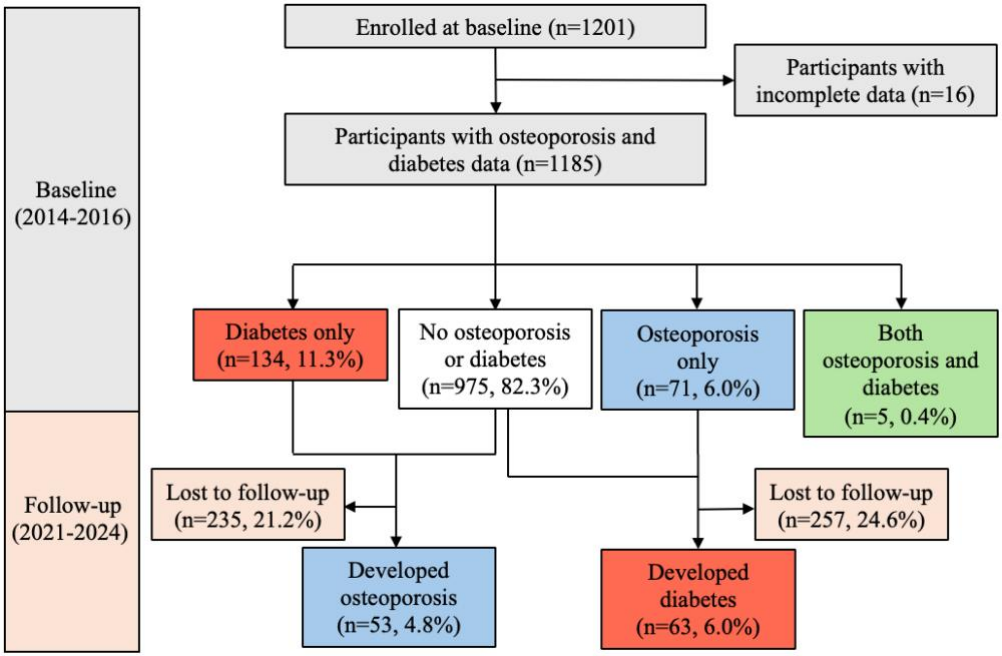
Participant flowchart detailing baseline and follow-up visits in the Integrated Women's Health Program longitudinal cohort.

The Integrated Woman's Health Program is a longitudinal cohort designed to examine critical health issues faced by midlife women. Details of the cohort have been previously described [22]. Baseline recruitment occurred between 2014 and 2016. Community-dwelling women (n = 1201, age 45-69 years) were enrolled from gynecological clinics at the National University Hospital, Singapore (Fig. 1). The participants were recontacted from 2021 to 2024 for follow-up assessments. Data on sociodemographics, medical history, medications, body composition, and biophysical metrics were collected at both visits. Ethical approval was obtained from the National Healthcare Group Domain Specific Review Board (reference nos. 2014/00356 and 2020/00201). All participants provided written informed consent.

### Data collection

At baseline, age, ethnicity, educational attainment, employment status, alcohol consumption, smoking status, as well as medical history were collected using standardized questionnaires. Trained study coordinators recorded the name, dosage, and frequency of medications and supplements consumed by participants in the previous 2 weeks. Information on the use of menopausal hormone therapy (MHT) was collected via a medication inventory.

Height was measured twice and weight once using a calibrated digital scale (SECA 769; SECA GmbH & Co. KG., Hamburg, Germany) by trained staff. BMI was calculated as weight (kg) divided by height-squared (m^2^) and classified using Asian-specific categories: <18.5 kg/m^2^ (underweight), 18.5 to 23 kg/m^2^ (normal), 23 to 27.5 kg/m^2^ (overweight), and ≥27.5 kg/m^2^ (obese) [23].

### Measurement of VAT

VAT, at both baseline and follow-up visits, was assessed using whole-body dual-energy X-ray absorptiometry (DXA) scanning using Hologic Discovery Wi (Hologic Inc., Bedford, MA, USA) with Apex software version 4.5. The software used algorithms to identify fat deposits at the level of the fourth lumbar vertebrae. With appropriate modeling, the amount of subcutaneous fat in the abdominal region was estimated from the subcutaneous fat on each side of the abdominal cavity. This estimate of subcutaneous fat was subtracted from the total abdominal fat in the abdominal region to yield VAT [11]. As a quality control tool, a whole-body phantom (Hologic, Inc.) was used regularly to calibrate the DXA scanner, ensuring instrument stability for measurements over the period of the study. Historically, VAT by computed tomography or magnetic resonance imaging scans are considered the gold-standard modalities [24]. Since VAT by DXA or computed tomography is correlated closely (r = 0.93), we elected to use DXA as this modality was more readily available in clinical care settings and more easily accessible for epidemiological studies [25, 26].

### Diabetes

Diabetes was assessed at both the baseline and follow-up visits and defined based on the American Diabetes Association criteria of fasting plasma glucose ≥7.0 mmol/L, self-reported use of diabetes medication, or physician-confirmed diagnosis [27]. We used a combination criteria including self-reports of physician-diagnosed diabetes or the initiation of glucose-lowering medications (insulin or oral agents) during follow-up to capture cases diagnosed in routine clinical practice outside of the study's specific testing schedule [27]. Fasting blood samples were collected for the determination of glucose concentrations following an overnight fast, and all samples were processed within 6 hours and stored at −80°C for batch analyses. To address missing data, electronic medical records were reviewed for participants lacking biochemical or medication data at follow-up. Incident diabetes was defined as cases with no diabetes at baseline but who developed the condition at the 6.8-year follow-up visit (Fig. 1).

### Osteoporosis

Osteoporosis was assessed at both baseline and follow-up visits and defined as a lumbar spine (vertebrae L1-L4) T-score of ≤−2.5 on DXA [28]. BMD at lumbar spines (vertebrae L1-L4) was measured using whole-body DXA (Hologic Discovery Wi, Hologic Inc.) scanning with Apex software version 4.5. Quality control and calibration were conducted in accordance with the manufacturer's protocols before each session. The lumbar spine was selected as the primary site for BMD measurement because it is a clinically sensitive region for detecting early bone loss, particularly in younger populations [28]. Incident osteoporosis was defined as cases with no osteoporosis at baseline but who developed the condition at the 6.8-year follow-up visit (Fig. 1).

### Statistical analyses

Descriptive statistics were reported according to baseline disease status: (1) no osteoporosis or diabetes, (2) osteoporosis only, (3) diabetes only, and (4) both osteoporosis and/or diabetes. Categorical variables were summarized as frequencies and percentages; group differences were assessed via Pearson's chi-squared test. Continuous variables were compared using 1-way ANOVA (parametric) or Kruskal–Wallis test (nonparametric) and expressed as means with SD or medians with interquartile ranges.

Receiver operating characteristic curves were used to evaluate the ability of BMI and VAT to discriminate osteoporosis and/or diabetes at baseline. Area under the curve (AUC) was computed for each measure.

Binary logistic regression with cubic spline terms (4 degrees of freedom) was applied to model nonlinear relationships between VAT and the disease risk. Extreme VAT values ≥220 cm^2^ were truncated at 220 cm^2^ to reduce outlier influence. Models were adjusted for age and ethnicity using mean cohort age and the most prevalent ethnicity for prediction. We did not adjust for menopausal status as this variable correlates highly with age and as such might result in overfitting in our models. Odds ratios (ORs) and 95% confidence intervals (CIs) were calculated by exponentiating the linear predictor from the fitted spline model. The optimal VAT value was defined as the point with the lowest predicted disease risk, while the healthy range was identified from where the OR curve intersected or approached 1.0. A rug plot was used to visualize VAT distribution.

Validation of VAT thresholds was conducted by examining incident osteoporosis and/or diabetes over 6.8 years using modified Poisson. Models were adjusted for age and ethnicity (selected due to <.05 in univariate analysis). Smoking was excluded due to low prevalence (<2%). BMI, fasting glucose, insulin, and BMD were not included due to high collinearity with VAT or the osteoporosis and/or diabetes. To assess the effect of attrition bias, a sensitivity analysis using inverse probability weighting was conducted for baseline characteristics that differed between the analytical sample and participants who were lost to follow-up for each incident disease. Sensitivity analyses were also conducted to account for the use of MHT at baseline. Statistical analyses were performed using SPSS version 29.0 (IBM Corp., Armonk, NY, USA) and R version 4.3.1with the ggplot2 for visualization.

## Results

### Participant characteristics

Of 1201 participants enrolled at baseline, 16 were excluded due to incomplete data, leaving 1185 (mean age: 56.3 ± 6.2, age range: 45.0-69.6 years) for analysis with data for both osteoporosis and/or diabetes (Fig. 1). Among the analytical sample of 1185 subjects, 12.6% were premenopausal, 15.7% were in perimenopause, and the majority (71.7%) were postmenopausal. The use of systemic MHT at baseline was low in our cohort at 6.2%. At baseline, 82.3% had neither osteoporosis nor diabetes, 11.3% had diabetes only, 6.0% had osteoporosis only, and 0.4% had osteoporosis and diabetes. Table 1 shows the characteristics of participants at baseline. Women with osteoporosis were older than those without (mean age: 59.4 ± 5.5 vs 55.9 ± 6.1 years). Diabetes prevalence was higher among Indian (25.2%) and Malay (18.2%) women than Chinese (9.0%). Conversely, osteoporosis prevalence was higher in Chinese (6.4%) and Malay (6.1%) women than in Indian women (3.4%). Smokers had twice the diabetes prevalence compared to nonsmokers (24.0% vs 11.1%).

**Table 1:** Baseline characteristics ⁠of participants with type 2 diabetes and spinal osteoporosis at baseline (n = 1185)

Baseline characteristics	No diabetes or osteoporosis (n = 975, 82.3%)	Diabetes only (n = 134, 11.3%)	Osteoporosis only (n = 71, 6.0%)	Both diabetes and osteoporosis (n = 5, 0.4%)	*P*-value
Age (years), mean ± SD	55.9 ± 6.1	57.3 ± 6.2	59.4 ± 5.5	60.9 ± 6.6	<.001
Ethnicity, n (%)					<.001
Chinese	809 (84.0)	87 (9.0)	62 (6.4)	5 (0.5)	
Malay	50 (75.8)	12 (18.2)	4 (6.1)	0 (0.0)	
Indian	85 (71.4)	30 (25.2)	4 (3.4)	0 (0.0)	
Education, n (%)					.295
No formal education or until primary school	132 (78.6)	20 (11.9)	15 (8.9)	1 (0.6)	
Secondary or postsecondary	624 (81.5)	94 (12.3)	44 (5.7)	4 (0.5)	
University	205 (86.5)	20 (8.4)	12 (5.1)	0 (0.0)	
Employment, n (%)					.090
Employed	661 (83.9)	84 (10.7)	39 (4.9)	4 (0.5)	
Not employed	309 (79.0)	49 (12.5)	32 (8.2)	1 (0.3)	
Alcohol, n (%)					.127
Drinker	67 (84.8)	4 (5.1)	8 (10.1)	0 (0.0)	
Nondrinker	904 (82.1)	129 (11.7)	63 (5.70)	5 (0.5)	
Smoking, n (%)					.004
Smoker	18 (72.0)	6 (24.0)	0 (0.0)	1 (4.0)	
Nonsmoker	952 (82.4)	128 (11.1)	71 (6.1)	4 (0.3)	
Menopausal hormone therapy, n (%)					.489
User	61 (83.6)	6 (8.2)	5 (6.8)	1 (1.4)	
Nonuser	914 (82.2)	128 (11.5)	66 (5.9)	4 (0.4)	
BMI kg/m^2^, n (%)					<.001
Underweight (<18.5)	44 (72.1)	2 (3.3)	15 (24.6)	0 (0.0)	
Normal (18.5-22.9)	423 (85.3)	32 (6.5)	38 (7.7)	3 (0.6)	
Overweight (23.0-27.4)	338 (83.5)	50 (12.3)	16 (4.0)	1 (0.2)	
Obese (≥27.5)	170 (76.2)	50 (22.4)	2 (0.9)	1 (0.4)	
VAT cm^2^, n (%)					<.001
<46.5	55 (79.7)	1 (1.4)	12 (17.4)	1 (1.4)	
46.5-116.6	499 (86.3)	31 (5.4)	46 (8.0)	2 (0.3)	
>116.6	412 (77.9)	102 (19.3)	13 (2.5)	2 (0.4)	
Fasting glucose mmol/L, median (IQR)	4.8 (0.7)	7.1 (2.4)	4.7 (0.5)	6.4 (2.1)	<.001
Insulin mIU/L, median (IQR)	4.9 (4.1)	8.2 (7.3)	3.4 (2.5)	7.3 (4.7)	<.001
Bone mineral density g/cm^2^, mean ± SD					
Lumbar spine	0.93 ± 0.13	0.99 ± 0.15	0.67 ± 0.05	0.67 ± 0.03	<.001
Femoral neck	0.71 ± 0.13	0.76 ± 0.14	0.57 ± 0.07	0.61 ± 0.12	<.001

Abbreviations: BMI, body mass index; IQR, interquartile range; VAT, visceral adipose tissue.

BMI distributions differed significantly across disease groups (*P* < .001). Obesity (BMI ≥27.5 kg/m^2^) was most common in the diabetes-only group (22.4%), while being underweight (BMI <18.5 kg/m^2^) was most frequent in the osteoporosis-only group (24.6%). These trends were mirrored by VAT patterns: high VAT (>116.6 cm^2^) was most prevalent in the diabetes-only group (19.3%), while low VAT (<46.5 cm^2^) was highest in the osteoporosis-only group (17.4%).

### Comparative performance of VAT and BMI for risk of osteoporosis and/or diabetes

**Figure 2:**
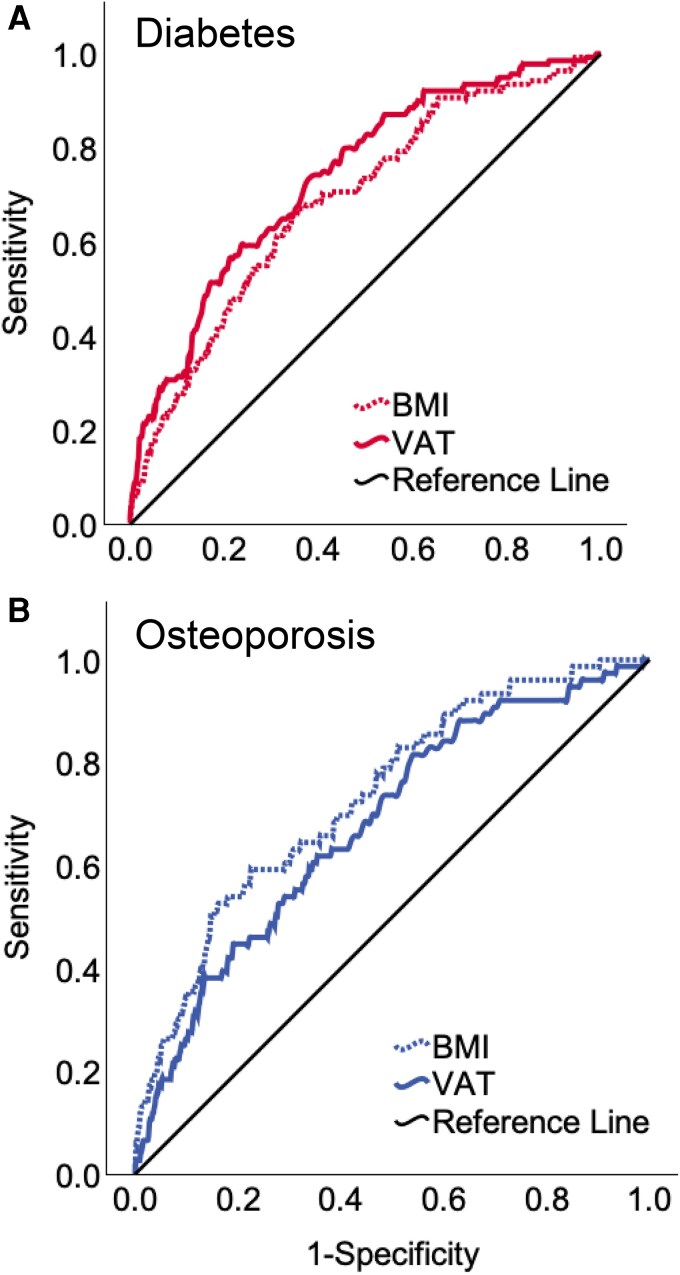
Receiver operating characteristic curves illustrating the discriminatory ability of body mass index and visceral adipose tissue (both at baseline) to identify (A) diabetes and (B) osteoporosis.

Receiver operating characteristic curves were constructed to compare VAT and BMI in predicting osteoporosis and/or diabetes (Fig. 2). Despite a strong correlation (r = 0.82), VAT outperformed BMI for predicting diabetes (Fig. 2A), with a higher AUC (0.739, 95% CI: 0.695-0.782) compared to BMI (0.687, 95% CI: 0.640-0.734), suggesting moderately better discriminative ability. For osteoporosis (Fig. 2B), BMI showed a slightly stronger discriminative ability with a higher AUC (0.734, 95% CI: 0.677-0.791) compared to VAT (0.680, 95% CI: 0.618-0.741), although the difference was not statistically significant (*P* = .089). Based on VAT's superior predictive ability for diabetes and its acceptable performance for osteoporosis [16, 29], we selected VAT as the primary adiposity marker for a combined risk of osteoporosis and/or diabetes.

### Associations between VAT and combined risk of diabetes and/or osteoporosis

**Figure 3:**
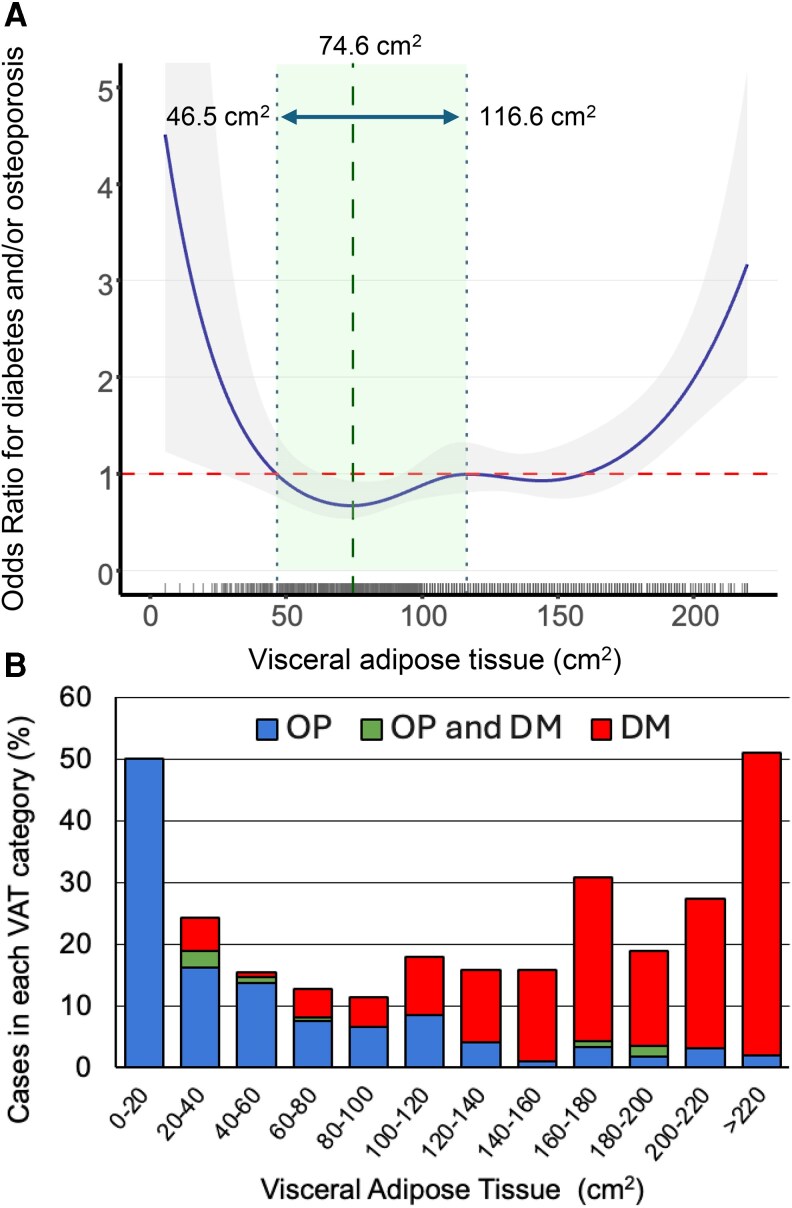
(A) Effect of VAT on the risk of diabetes and/or osteoporosis. The spline curve shows the odds ratio (95% confidence interval) of having diabetes and/or osteoporosis at specific VAT values, adjusted for age and ethnicity. The vertical green dotted line indicates the VAT value (74.6 cm^2^) associated with the lowest risk. Horizontal green arrows indicate the optimal VAT range (46.5-116.6 cm^2^) for the lowest risk (odds ratio ≤ 1) for diabetes and/or osteoporosis. The rug plot along the *X*-axis shows the distribution of observed VAT values. (B) Bar chart shows the distribution of diabetes and/or osteoporosis cases across VAT categories. Abbreviations: VAT, visceral adipose tissue.

We used spline regression models, adjusted for age and ethnicity, to examine the relationship between VAT and combined risk of diabetes and/or osteoporosis. The spline curve revealed a U-shaped association between VAT and combined risk of osteoporosis and/or diabetes (Fig. 3A). Both low and high VAT values were associated with increased risk, while the intermediate VAT range of 46.5 to 116.6 cm^2^ was associated with the lowest combined disease risk, wherein OR was ≤1.0. Disease prevalence by VAT categories is illustrated in Fig. 3B. Osteoporosis cases were clustered in the lower VAT categories (≤40.0 cm^2^), while diabetes cases were in the highest VAT categories (≥100 cm^2^). Women within the optimal VAT range (46.5-116.6 cm^2^) had the lowest combined disease risk at 13.7% (Table 1). We therefore selected 46.5 and 116.6 cm^2^ as clinically relevant lower and upper thresholds of VAT that minimizes combined metabolic and skeletal risk.

### Associations between VAT thresholds and risk of incident (new) osteoporosis and/or diabetes

We applied the derived VAT thresholds (<46.5, 46.5-116.6, and >116.6 cm^2^) to examine the risk of incident osteoporosis and incident diabetes that developed at the 6.8-year follow-up visit among those who did not have the disease at baseline (Fig. 1). Among 1109 participants without osteoporosis at baseline, 53 (4.8%) developed osteoporosis at follow-up. Among 1046 without diabetes at baseline, 63 (6.0%) developed diabetes at follow-up. For incident osteoporosis, participants who were lost to follow-up were more likely to be of lower education levels, be unemployed, smoke, and have higher VAT (Table S1) [30]. For incident diabetes, participants who were lost to follow-up were more likely to be of lower education levels and be unemployed (Table S2) [30]. Compared to those in the healthy VAT range, participants with high VAT (>116.6 cm^2^) had an increased risk of incident diabetes [adjusted relative risk (aRR): 2.89, 95% CI: 1.68-4.98, *P* < .001], and reduced osteoporosis risk (aRR: 0.33, 95% CI: 0.17-0.64, *P* = .001), after accounting for age and ethnicity (Table 2). In contrast, the low VAT group (<46.5 cm^2^) had no incident diabetes cases but showed a near-doubling of osteoporosis risk (aRR: 2.04, 95% CI: 0.95-4.40, *P* = .067). Sensitivity analyses accounting for attrition and baseline MHT use yielded similar results (Table S3 and S4) [30].

**Table 2:** Poisson regression analyses for associations between baseline VAT and the development of incident osteoporosis and incident diabetes at 6.8-year follow-up visit of the IWHP cohort

VAT thresholds, cm^2^	Incident osteoporosis			Incident diabetes		
	aRR (95% CI)	*P*-value	n	aRR (95% CI)	*P*-value	n
<46.5	2.04 (0.95, 4.40)	.067	8	—	—	0
46.5-116.6	Reference		34	Reference		17
>116.6	0.33 (0.17, 0.64)	<.001	10	2.89 (1.68, 4.98)	<.001	45

Results are presented as aRR with 95% CI, controlling for age and ethnicity. — indicates no cases of incident diabetes were observed for VAT <46.5 cm^2^.

Abbreviations: aRR, adjusted relative risk; CI, confidence interval; IWHP, Integrated Women's Health Programme; VAT, visceral adipose tissue.

## Discussion

Although the contribution of VAT to metabolic diseases has been increasingly recognized, no study, to our knowledge, has defined clinically relevant VAT thresholds [25]. In line with our hypothesis, we identified a healthy VAT range of 46.5 to 116.6 cm^2^ that was associated with the lowest risk for both osteoporosis and/or diabetes with a combined OR ≤1.0. This range was validated using new (incident) disease cases that developed on longitudinal follow-up. Using the healthy VAT range of 46.5 to 116.6 cm^2^ as reference, VAT < 46.5 cm^2^ was associated with a 2-fold increased risk of incident osteoporosis, while VAT > 116.6 cm^2^ increased incident diabetes risk by 189% and concurrently reduced incident osteoporosis risk by 67%. These findings support the validity of the optimal VAT range of 46.5 to 116.6 cm^2^ for the lowest risk for both osteoporosis and/or diabetes.

Since VAT is a better predictor of type 2 diabetes than general obesity and contributes to diabetes risk even in healthy, nonobese women [31], attempts have been made to define upper thresholds of VAT for risk stratification with respect to diabetes and metabolic syndrome [25]. The VAT threshold for diabetes from our study was 116.6 cm^2^, consistent with a review of 52 studies indicating VAT threshold ranges between 70 and 165.9 cm^2^ for diabetes and metabolic syndrome [25]. Our study for the first time identified an upper VAT threshold that would be relevant for clinicians managing diabetic risks in Singaporean midlife women, since thresholds do vary depending on age, sex, ethnicity, and geographical location.

To determine the lower threshold for VAT, we exploited the association of low VAT with reduced BMD. Although low levels of VAT have been associated with lower BMD in Taiwanese [32], UK [18], Australian [33], US [19], and Brazilian [34] populations, a lower threshold for healthy VAT has not been established. To our knowledge, this is the first study to report a lower threshold for VAT and to identify 46.5 cm^2^ as the lower threshold that minimizes risk for diabetes and osteoporosis. We then went on to validate these thresholds on risk for the development of (new) incident diabetes and incident osteoporosis cases using longitudinal follow-up data.

Our findings are consistent with known pathophysiological mechanisms. High VAT increases diabetes risk, likely mediated by insulin resistance, chronic inflammation, and lipotoxicity-induced β-cell dysfunction [20, 35]. In contrast, low VAT may impair bone health due to reduced mechanical loading and diminished leptin and estrogenic support, altering bone marrow adipogenesis [15]. Importantly, our findings were validated prospectively: baseline VAT outside the optimal range predicted future disease. VAT > 116.6 cm^2^ increased diabetes risk and reduced osteoporosis risk, while VAT <46.5 cm^2^ elevated osteoporosis risk. These opposing trends support a healthy VAT range as a clinically relevant threshold. Moreover, DXA-based VAT assessment is easily available and offers greater specificity than surrogate markers like waist circumference [36]. Availability of these validated VAT thresholds would be useful to healthcare professionals assessing combined risks for diabetes and osteoporosis in midlife women and serve as a target for behavioral interventions such as diet, physical activity, and chemoprevention.

Our findings indicate the need to move beyond BMI as an indicator of risk for diabetes and osteoporosis [37]. A Korean study identified a BMI range of 23.0 to 24.9 kg/m^2^ as optimal for minimizing the risk of osteoporosis and diabetes [38]. However, traditional reliance on BMI fails to capture nuances in ectopic fat distribution, especially metabolic dysfunction associated with high VAT [25, 35]. In addition, it is not commonly appreciated that high BMI could also be due to the contribution of high skeletal muscle mass, a metabolically active tissue that reduces diabetes risk [36, 39]. Furthermore, individuals with elevated BMI are not uniformly metabolically unhealthy—especially those with subcutaneous or gluteo-femoral fat predominance [40]. Conversely, individuals with BMI in the normal range but with elevated VAT may be at disproportionately high metabolic risk—a phenotype increasingly recognized in Asian populations [1, 20]. Our findings support a shift toward VAT-based risk stratification, particularly for Asian women, among whom “metabolically obese, normal-weight” phenotypes are common [11].

Our findings indicate the important role of VAT for energy homeostasis. VAT is the primary depot for storage of excess energy and its rapid mobilization in fasting states. In times of chronic caloric overabundance, VAT storage and size can increase excessively, inducing changes to regulatory T cells and leading to increased inflammatory cytokines and higher diabetes risk [41]. On the other hand, our study for the first time also indicates a lower threshold for VAT, below which an increased risk for osteoporosis can be observed. The mechanistic basis whereby low VAT results in osteoporosis is still unclear. Nevertheless, an informative model might be the state of chronic undernutrition and starvation in anorexia nervosa, where low VAT was paradoxically associated with elevated bone marrow adipose tissue [42, 43]. Bone marrow adipose tissue is an independent driver for bone loss [44], and a causal link between bone marrow adipose tissue and osteoporosis has been shown by Mendelian randomization analyses in UK Biobank participants [45]. In underweight subjects, interventions to prevent bone loss might include consumption of fish oil (rich in ω3 fatty acids) which has been shown to reduce bone marrow adipose tissue in animal models [46]. In obese subjects, weight loss plus a combination of aerobic and resistance exercise can improve ectopic fat deposition and physical and metabolic function [47, 48]. In totality, our findings suggest the need for a balance between calorie intake and physical activity for optimal VAT homeostasis and the lowest risk of diabetes and osteoporosis.

This study had several limitations. Our study population included only midlife Singaporean women of Chinese, Malay, and Indian ethnicities, which may limit generalizability to other age groups, ethnicities, and geographical locations. The VAT thresholds identified in our study would need to be revalidated in other populations, as published data on upper thresholds relevant to risk for diabetes and metabolic syndrome indicate a fluctuating range depending on age, sex, ethnicity, and geographical location [25 ]. Nonetheless, our models were adjusted for age and ethnicity, allowing comparison with published thresholds for the risk of diabetes [25]. Participants were recruited from a single tertiary hospital, and although the cohort was ethnically diverse and broadly representative of Singapore's population, external validity to the rest of the country may still be constrained. Furthermore, while DXA measured VAT accurately, it did not capture other ectopic fat sites (eg, liver, pancreas, skeletal muscle) that may influence disease risk. The cases lost to follow-up after a mean follow-up period of 6.8 years was 21% to 24% (Fig. 1), an attrition rate that may be considered high. However, sensitivity analyses accounting for attrition and baseline MHT use yielded similar results and indicated that the conclusions remained unchanged (Table S3 and S4) [30]. Finally, we did not evaluate whether VAT-targeting exercise interventions could shift individuals into the healthy VAT range and reduce long-term disease outcomes—an area for future interventional research [49, 50].

Despite these limitations, our study offers several strengths that distinguishes it from existing research. Unlike prior studies that focused on BMI, we defined direct VAT thresholds for osteoporosis and/or diabetes in a multiethnic Asian population. Additionally, our study utilized direct DXA-based VAT, which provided precise quantification, and the findings were validated over a 6.8-year follow-up. The inclusion of 3 major ethnic groups (Chinese, Malay, Indian) reflected nearly 50% of the global female population. The use of standardized diagnostic criteria and adjustment for confounders further enhanced the scientific rigor of our findings.

### Conclusion

Menopause is associated with bone loss and enhanced visceral adiposity [50-53]. Our study provided novel insights about the dual role of VAT in influencing the risk of osteoporosis and diabetes in postmenopausal women. We identified an optimal VAT range of 46.5 to 116.6 cm^2^ that was associated with the lowest combined disease risk and have validated it against incident disease. This range can be used to guide individualized risk stratification and preventive care, particularly in Asian populations where high VAT and normal BMI are common. Our findings have increasing relevance with the use of current VAT-targeting treatments such as glucagon-like peptide-1 receptor agonists, which have demonstrated VAT-reducing efficacy and broad cardiometabolic benefits [13]. Future research should assess whether lifestyle, or pharmacologic interventions such as FSH-blocking monoclonal antibodies, can lower visceral fat [54] to a healthy VAT range, with lowest risk for diabetes and osteoporosis.

## Data Availability

Data cannot be shared publicly due to ethical-legal reasons, and consent was not sought from study participants for public data sharing. Data is available from the corresponding author at obgyel@nus.edu.sg upon reasonable request and for researchers who meet the criteria for access to confidential data.
